# Individual Alpha Peak Frequency, an Important Biomarker for Live Z-Score Training Neurofeedback in Adolescents with Learning Disabilities

**DOI:** 10.3390/brainsci11020167

**Published:** 2021-01-28

**Authors:** Rubén Pérez-Elvira, Javier Oltra-Cucarella, José Antonio Carrobles, Minodora Teodoru, Ciprian Bacila, Bogdan Neamtu

**Affiliations:** 1 Neuropsychophysiology lab., NEPSA Rehabilitación Neurológica, 37003 Salamanca, Spain; rubenperezelvira@gmail.com; 2 Department of Health Psychology, Universidad Miguel Hernández de Elche, 03202 Elche, Spain; 3 Biological and Health Psychology Department, Universidad Autónoma de Madrid, 28049 Madrid, Spain; joseantonio.carrobles@uam.es; 4 Faculty of Medicine, Lucian Blaga University from Sibiu, 550169 Sibiu, Romania; minodora.teodoru@ulbsibiu.ro (M.T.); bacila_c@yahoo.com (C.B.); bogdan.neamtu@ulbsibiu.ro (B.N.); 5 “Dr. Gheorghe Preda” Psychiatric Hospital of Sibiu, 550082 Sibiu, Romania; 6 Research Department (Ceforaten), Sibiu Pediatric Hospital, 550178 Sibiu, Romania; 7 Faculty of Engineering, Lucian Blaga University from Sibiu, 550025 Sibiu, Romania

**Keywords:** neurofeedback, Z-score training, learning disabilities, endophenotypes, alpha peak frequency, QEEG

## Abstract

Learning disabilities (LDs) have an estimated prevalence between 5% and 9% in the pediatric population and are associated with difficulties in reading, arithmetic, and writing. Previous electroencephalography (EEG) research has reported a lag in alpha-band development in specific LD phenotypes, which seems to offer a possible explanation for differences in EEG maturation. In this study, 40 adolescents aged 10–15 years with LDs underwent 10 sessions of Live Z-Score Training Neurofeedback (LZT-NF) Training to improve their cognition and behavior. Based on the individual alpha peak frequency (i-APF) values from the spectrogram, a group with normal i-APF (ni-APF) and a group with low i-APF (li-APF) were compared in a pre-and-post-LZT-NF intervention. There were no statistical differences in age, gender, or the distribution of LDs between the groups. The li-APF group showed a higher theta absolute power in P4 (*p* = 0.016) at baseline and higher Hi-Beta absolute power in F3 (*p* = 0.007) post-treatment compared with the ni-APF group. In both groups, extreme waves (absolute Z-score of ≥1.5) were more likely to move toward the normative values, with better results in the ni-APF group. Conversely, the waves within the normal range at baseline were more likely to move out of the range after treatment in the li-APF group. Our results provide evidence of a viable biomarker for identifying optimal responders for the LZT-NF technique based on the i-APF metric reflecting the patient’s neurophysiological individuality.

## 1. Introduction

Learning disabilities (LDs) have an estimated prevalence of 5–9% in pediatric populations, with a higher incidence in boys than in girls (up to 9:1) [1]. According to the American Psychiatric Association [2], LDs are diagnosed based on significantly lower performance in one or more tests measuring reading, arithmetic, or writing [2,3]. Abnormal electroencephalography (EEG) patterns in children and adolescents with LDs have previously been reported [3,4]. EEG and quantitative EEG (QEEG) provide useful insights in these cases regarding the brain’s electrical function, revealing slower activity, especially in the alpha and theta bands, compared with that in age-matched typically developing children [3,5]. Low cognitive performance in children and adolescents with LDs seems to be related to a deviation from normal neural network development manifesting as an alpha-band developmental lag, which seems to explain differences in EEG maturation found in children and adolescents with this condition [3,6,7,8,9,10]. A viable candidate for an LDs biomarker based on the alpha band is the individual alpha peak frequency (i-APF), a discrete frequency at which alpha waves acquire their highest amplitude [11,12,13,14], mainly occurring in the posterior regions of the scalp and in closed-eye conditions [15]. The normal values of i-APF are age related; a mature alpha frequency of 10 Hz is commonly reached by 10 years of age, while the maximum alpha peak is reached before this age [16,17]. It is acknowledged as an endophenotype, highly stable across time for each subject and highly sensitive to developmental changes in cognitive neural networks, with its variance among individuals depending on the genotype [17,18,19,20]. Research reports suggest that the i-APF is generated by thalamocortical feedback loops reflecting the speed of information processing. Therefore, the i-APF might be a useful biomarker for the cerebral cortex’s ability to poll information from the thalamus and to relay back that information to the thalamus. These are important processes for working and semantic memory [17,18,19,20].

Moreover, the i-APF might be considered a feature-like EEG biomarker, as it correlates with individual differences in cognitive performance [17,19]. Normal i-APF values are common in healthy children, while some children and adolescents with LDs, autism spectrum disorders (ASDs), or attention-deficit/hyperactivity disorder (ADHD) show phenotypes with low i-APFs (<9 Hz), and some cases with this phenotype can be classified as nonresponders to different treatments (such as pharmacological treatments, repetitive transcranial magnetic stimulation (rTMS), and neurotherapy) [10,12,21,22].

There is an urgent need to develop new methods for LD treatment, including those based on neurotherapy (e.g., neurofeedback) [12,13,14,15]. Neurofeedback (NF) is an electroencephalographic technique that uses operant conditioning to train, in a nonvoluntary manner, the subject’s brain activity in terms of EEG metrics (power, amplitude, coherence, and phase) to modulate it towards the normative data in the QEEG database, while the subject receives different visual and/or auditory stimuli (video games and movies) [11,15,23,24]. Real-time NF techniques have shown promise in improving the cognitive performance of patients with LDs, ADHD, and ASD [24,25,26,27,28,29,30]. However, the brain activity metrics in some of these patients (nonresponders) do not seem to improve, even after having several sessions [24,25,26,27,28,29,30] or applying advanced, novel self-regulation training techniques, such as Live Z-Score Training Neurofeedback (LZT-NF). LZT-NF performs real-time QEEG in the form of generating Z-scores as an essential component of the feedback control mechanism. It combines different EEG metrics (power, amplitude, coherence, and phase) into a single category of metrics, the Z-scores, to compare the values of the studied subjects and the reference values of the age-matched healthy patients documented in the normative databases [31,32,33,34,35,36]. Despite its potential beneficial effects, namely, fewer sessions needed to meet the goals in patients responding to the neurofeedback approach, in ADHD, for example, not all subjects respond well to LZT-NF interventions [24,37,38,39]. This suggests that some specific factors related to each individual might be the moderators of a more successful response [40]. A higher working memory, better attentional resources, better learning skills, better mood, personality variables, or association of the internal locus of control reinforcement with the EEG control seem to be crucial in this respect [25,41,42,43,44,45,46,47]. Some authors have proposed the i-APF as a forecasting factor for the subject’s capacity to modulate the EEG data for teenagers and adults [48,49]. However, the literature on the factors leading individuals with LDs to not respond to NF is scarce [4,21,40,50].

The aim of our study was to explore potential EEG-based biomarkers of LDs and to guide LZT-NF interventions using information obtained during tests with EEG markers of the condition. According to some reports in the literature, a mature alpha frequency of 10 Hz may be reached within a larger age interval, 10–15 years, in normal children [20,51], while in ADHD children with impaired learning, frequencies below 9 Hz are considered biomarkers for slow alpha peak frequency according to Arns et al. [22]. Data on children with LDs are scarce in this respect, as are LZT-NF approaches for improving their cognitive functions. To meet our goal, we hypothesized that the i-APF, in particular, might be considered a moderator of QEEG normalization after LZT-NF intervention in adolescents aged 10–15 years with LDs. Moreover, we proposed a 9.5 Hz cutoff point value for the i-APF [13], which might guide future research approaches to classifying these patients based on i-APF categories (normal and low) and according to response (or lack thereof) to LZT-NF intervention. To test our hypothesis, we explored the LZT-NF response in a 4-out-of-19-channels (F3, F4, P3, and P4) QEEG based on i-APF categories (normal and low) in adolescents aged 10–15 years with LDs. We also investigated the feasibility of using only 10 sessions of LZT-NF with personalized reinforcers (different movies).

## 2. Materials and Methods

### 2.1. Participants

This work was conducted in NEPSA Rehabilitación Neurológica, a neurological rehabilitation clinic certified by the Government of Castilla y León (Spain), in collaboration with the Research and Telemedicine Center for Neurological Diseases in Children in Sibiu, Romania. Forty-five adolescents with LDs were enrolled between September 2017 and December 2019 to receive LZT-NF.

**Figure 1:**
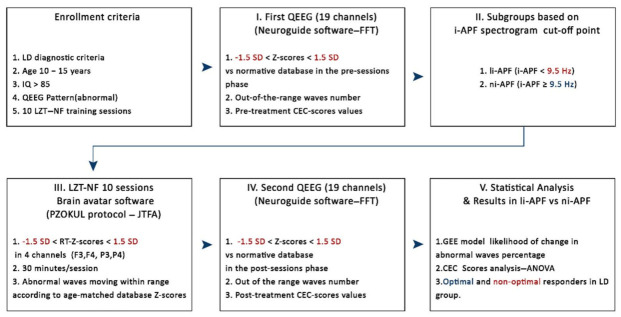
Enrollment criteria: I, first quantitative EEG (QEEG) to evaluate abnormal patterns vs. database norms and to compute out-of-the-range (±1.5 SD) waves number and Cognitive and Emotional Checklist (CEC) score values pre-LZT-NF (Live Z-Score Training Neurofeedback) sessions; II, li-APF (low individual alpha peak) and ni-APF (normal individual alpha peak) subgroup designation based on a 9.5 Hz cutoff point for i-APF (individual alpha peak visually identified in the spectrogram); III, 10 LZT-NF sessions (30 min each) with real-time (RT) Z-scores vs. database norms to constrain within the range (±1.5 SD) the abnormal waves; IV, second QEEG to evaluate abnormal patterns vs. database norms and to compute out-of-the-range (±1.5 SD) waves number and CEC score values post-LZT-NF sessions; V, statistical analysis in li-APF subjects regarding out-of-the-range waves number (±1.5 SD), before and after employing GEE (generalized estimating equation), and CEC scores using repeated measures ANOVA. LD: learning disabilities, FFT: Fast Fourier Transform, JTFA: Join Time Frequency Analysis, PZOKUL: BrainMaster protocol Percentage of Z-Scores OK Upper and Lower thresholds.

The subjects were selected based on specific criteria [21]: (1) being diagnosed with LDs by a team consensus among school psychologists and neuropediatricians and clinical psychologists from our clinic, according to both DSM-5 [2] guidelines and the government criteria for the classification of LDs in childhood (Instrucción de 24 August 2017 de la Consejería de Educación de la Junta de Castilla y León, Spain) [52]; (2) being aged 10–15 years; (3) having an intelligence quotient (IQ) higher than 85 according to the Wechsler Intelligence Scale for Children, 4th ed. [53]; (4) having a QEEG pattern with multiple abnormal Z-scores (i.e., more than one abnormal wave in more than one location or region)—we considered a “low-voltage profile, increased generalized slowing, increased fast frequencies, high amplitude, atypical alpha, excess focal delta or theta, and persistent asymmetries” as suggested by Bosch-Bayard et al. [3], Chiarenza [5], and Fernández et al. [21]; and (5) having at least 10 Live Z-Score Training Neurofeedback (LZT-NF) technique sessions in the F3, F4, P3, and P4 locations. We excluded participants with (1) paroxysmal activity in every EEG frequency band [21]; (2) a history of any neurological or psychiatric disorder other than LDs, either as a single medical condition or in association with LDs; or (3) missing data for any of the main outcomes. The research methodology is presented in Figure 1.

### 2.2. Cognitive and Emotional Checklist

An experienced neuropsychologist interviewed the participants’ parents (mothers and/or fathers) or legal tutors using the Cognitive and Emotional Checklist (CEC), an inventory created by Soutar [54] to collect information about emotional, cognitive, and behavioral symptoms and monitor changes at follow-up. The interview’s main objective was to record the parents’ qualitative observations of behaviors before and after LZT-NF treatment regarding learning problems and difficulties with attention, memory, attitude, social interaction, and emotional changes.

The CEC includes 49 items that are answered by parents on a 4-point Likert-type scale ranging from 0 (no symptoms) to 3 (present and severe symptoms). The scores can range between 0 and 147, with higher scores indicating more severe symptoms. To assess the efficacy of the intervention against educational impairments, the 10 CEC items related to learning, mathematics, reading, and writing (CEC-Learning) were analyzed as separate outcomes. These items are presented in Appendix A, Table A1. For the rest of the CECs, the sum of the scores for these items ranges from 0 to 30, with higher scores indicating problems of greater frequency and severity. The main outcomes were the CEC-Total score and the CEC-Learning score.

### 2.3. EEG Collection and QEEG Analysis

**Figure 2:**
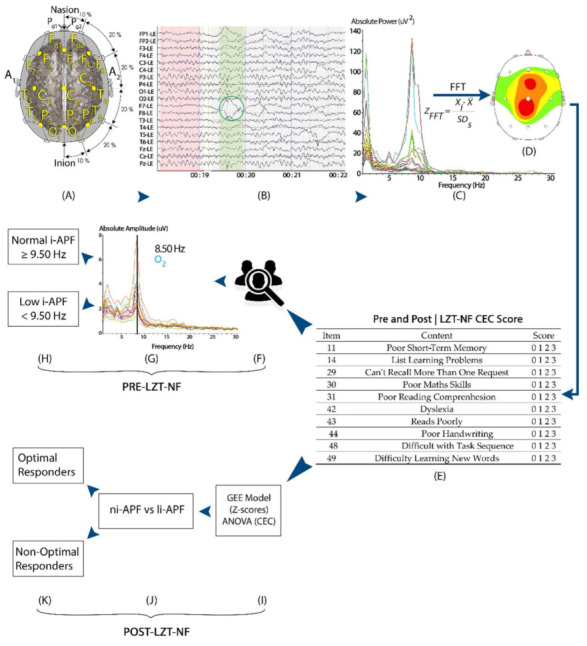
A visual representation of the study methods: participants’ EEG was measured with a 19-channel amplifier and a Linked Ears montage pre- and post-LZT-NF intervention (**A**–**C**). EEG from all 19 channels were imported and visually edited in NeuroGuide to remove artifacts (green circle) (**B**), and the fast Fourier transform converted the signal into frequency-based measures of absolute power and Z-scores (**C**,**D**). Participants’ parents/tutors filled the CEC both pre- and post-LZT-NF intervention (**E**). Participants were then divided into li-APF and ni-APF based on i-APF spectrogram pre-LZT-NF intervention (**F**–**H**). Post-LZT-NF intervention, repeated measures ANOVA and binary logistic regression analyzed the difference between ni-APF and li-APF groups to identify optimal responders (**I**–**K**).

Potential candidates were enrolled based on multiple abnormal Z-scores in more than one location and more than one frequency band. Abnormal Z-scores were defined as absolute Z-scores equal to or higher than 1.5. For the collection of the EEGs, the subjects were seated in a comfortable recliner, and each patient was fitted with an electroencephalography cap, the Electro-Cap (Electro-Cap International), with the 19 channels arranged according to the International 10–20 System (Fp1, Fp2, F7, F3, Fz, F4, F8, T3, C3, Cz, C4, T4, T5, P3, Pz, P4, T6, O1, and O2) and using a Linked Ears (LE) montage (Figure 2). The EEG data sampling rate was 256 samples/second. For 3–5 min, EEG signals from all 19 channels were simultaneously obtained and collected using a Discovery 20 amplifier (BrainMaster Technologies, Inc., Bedford, OH, USA). Impedances of less than 5 kOhms were maintained. EEG signals were recorded using BrainAvatar 4.6.4 (BrainMaster Technologies, Inc.). The EEG amplifier was set to a bandpass of 0.5 to 50 Hz [3,24].

The EEG records were imported into NeuroGuide v. 2.9.1 (Applied Neuroscience, Inc., St. Petersburg, FL, USA) for computation and analysis. An expert in QEEG analysis (certified by the Biofeedback Certification International Alliance) visually edited the EEG data to select at least 30 s of EEG segments free of artifacts for each subject to meet the conditions in the normative database embedded in the software for further data processing. On average, there were 1400 s of artifact-free data for the pre-treatment and post-treatment periods for our subjects [24].

We applied fast Fourier transform at every 10–20 System location to convert the signal into frequency-based measures of absolute and relative power in the classical frequency bands and 1 Hz bins for quantitative analysis [3,24]. Relative power was excluded, as it was a calculation of the absolute power distribution of the entire spectrogram. The NeuroGuide software automatically computes the absolute power, expressing its variations from the norms in terms of Z-scores (standard deviations compared with the mean) in seven frequency bands (Delta, 1–4Hz; Theta, 4–8 Hz; Alpha, 8–12 Hz; Beta-1, 12–15 Hz; Beta-2, 15–18 Hz; Beta-3, 18–25 Hz; and Hi-Beta, 25–30 Hz). The beta frequency was excluded because its activity was already included in the breakdown (Beta-1, Beta-2, and Beta-3); redundant data were therefore avoided [55], allowing each wave to be treated as a variable independent from the rest of the variables. The Z-scores were calculated for each frequency band at each location. We used color-coded brain maps to visualize the Z-scores, the values for each subject, and the values for each frequency band, with a focus on the abnormal Z-scores to be addressed [24].

**Figure 3:**
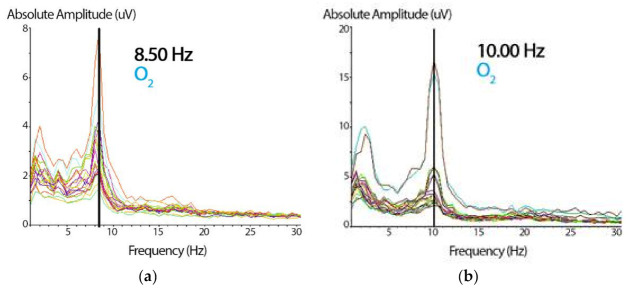
(**a**) Spectrogram showing the absolute amplitude peak representing the i-APF of the subject. Based on the cutoff point, these data (8.50 Hz) correspond to a participant with a low i-APF (in the right occipital, O_2_, from the 19-channel spectrogram). On the *x*-axis, the frequency is expressed in Hz, and on the *y*-axis, the wave’s absolute amplitude is expressed in microvolts (uV). (**b**) The same spectrogram shows the absolute amplitude peak with 10 Hz for a participant with a normal i-APF (the same right occipital, O_2_, channel). The abscissa and the ordinate parameters are similar to those presented in Figure 3a.

Once the sample’s artifacts were removed manually using the deletion method (selecting and deleting the artifacts), the spectrogram was examined. We used the Klimesch approach (Figure 3) and visually selected, from the 7.5–12.5 Hz band range, the peak frequency showing the largest power estimate within the spectral component to identify each subject’s i-APF [13]. According to the age-matched i-APF cutoff points reported by Klimesch et al. [13], Arns et al. [56], Blum and Rutkove [16], Holmes et al. [51], and Rubin and Daube [57], the participants were classified as being within the normal limits (ni-APF) when the i-APF values from the spectrogram at baseline were equal to or higher than 9.50 Hz, and as li-APF (li-APF) otherwise.

### 2.4. Neurofeedback Intervention (Live Z-Score Training Neurofeedback)

**Figure 4:**
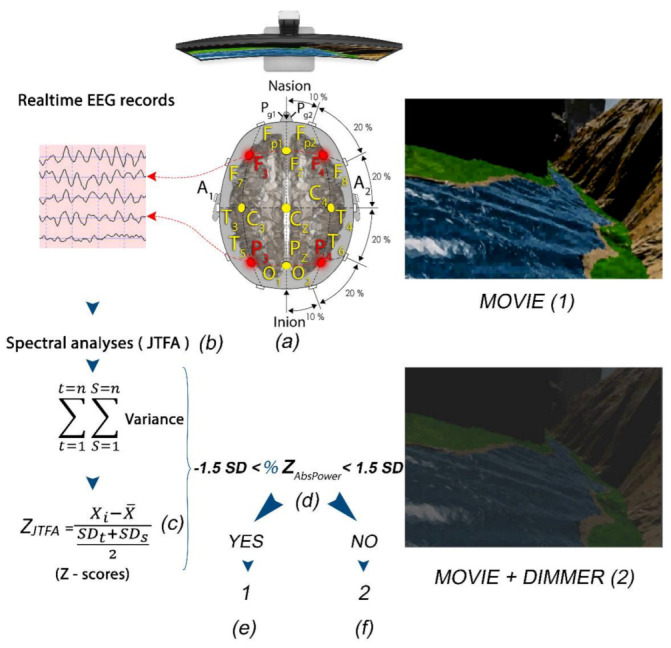
(**a**) Subject with F3, F4, P3, and P4 locations selected for LZT-NF protocol (PZOKUL) and LE montage. (**b**) Real-time EEG records from the four leads with Join Time Frequency Analysis (JTFA). (**c**) Z-scores (using JTFA) computed in real time. (**d**) %Z absolute power within ±1.5 SD. (**f**) If the %Z absolute power is within the range, then the display shows a movie with a clear image (1). (**e**) If the %Z absolute power is out of the range, then the display shows a movie with a dimmer that darkens the image (2).

The Z-score LZT-NF technique trained the oscillatory activity of the studied subjects by comparing their metrics (power, amplitude, coherence, and phase) with a normative database used as a reference. The reference is based on a repository of QEEG data from healthy persons age-matched with the trained subjects [31,32,33,34,35,36]. The Z-scores for any of the computed metrics were directly related to the numbers of standard deviations the values of the studied parameters were from the mean values for a subject’s reference groups [24]. The Z-scores were computed using joint time–frequency analysis (JTFA), which maps a one-dimensional time domain signal into a two-dimensional representation of energy versus time and frequency. The LZT-NF synchronously trains multiple metrics’ Z-scores to the center of the age-matched reference group’s Z-scores in real time (Figure 4).

In our study, a QEEG-guided LZT-NF protocol from BrainMaster Technologies, Inc. (LZT Percentage of Z-Score OK Upper and Lower thresholds, PZOKUL), was used. We selected F3, F4, P3, and P4 leads for channel modulation because of their capacity for global normalization (the red leads in Figure 4) [24]. The same LE montage was used with the ground in Cz. The mentioned protocol has a threshold for the percentage of Z-scores for absolute power that must fall within the established deviation range (set as −1.5 to 1.5), a threshold that is self-adjusted. This self-adjustment was based on the percentage of Z-scores for all the bands receiving treatment that fell within the set deviation range, with an upper threshold (positive Z-scores) and a lower threshold (negative Z-scores), and the percentage of reinforcement that the patient was achieving. The waves with Z-scores higher than ±1.5 SD were categorized as out of the range and were further analyzed in terms of absolute values [31,33].

According to the number of NF training sessions and session duration reported in previous works [24,39], including some of our own group [58,59], the patients underwent 10 30 min sessions delivered twice a week without interruption. The participants were allowed to choose both the form (i.e., visual/auditory) and feedback type in each session based on previous studies, indicating that the more relevant the enhancer was to the subject, the greater the learning effect was [60,61,62]. In all the cases, the selected enhancers were different movies (visual and auditory stimuli) preferred by each participant to ensure a personalized reinforcer [60,63].

**Figure A1:**
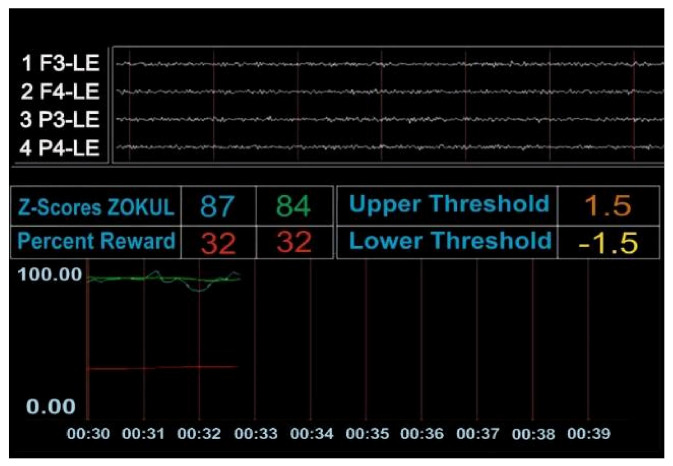

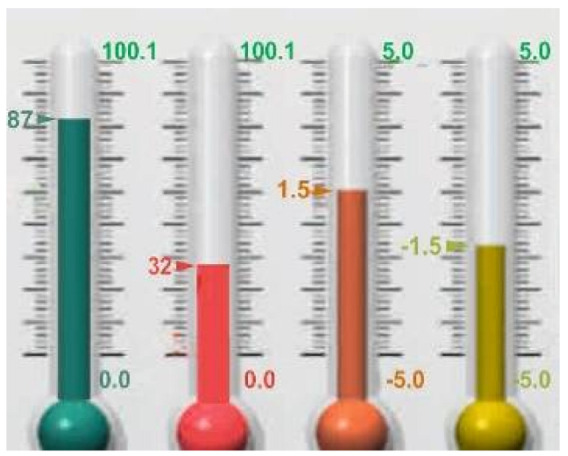
The patient achieved the percentage of Z-scores within the range (the number 87 in blue represents the percentage of Z values the patient was getting) to get the reinforcer (the number 84 in green represents the percentage of Z values that the patient was asked to put within the range to get the reinforcer).

**Figure A2:**
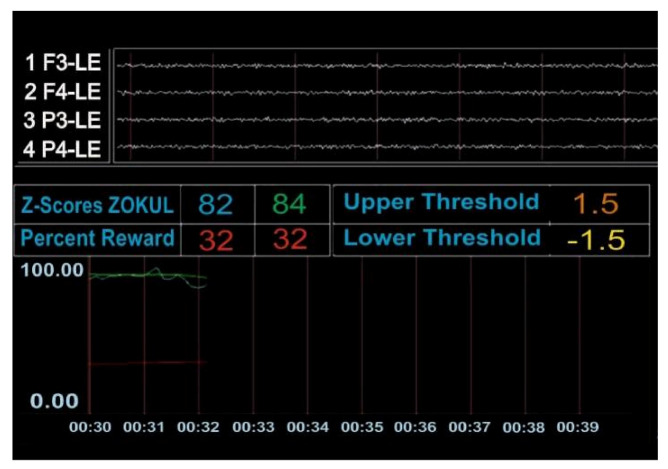

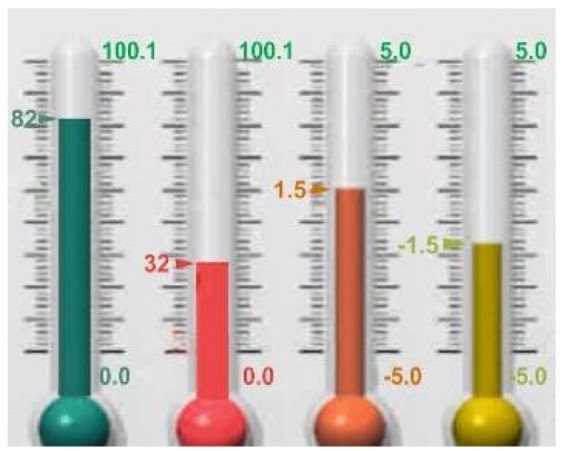
The patient was not achieving the percentage of Z-scores within the range (the number 82 in blue represents Table 84. in green represents the percentage of Z values that the patient was asked to put within the range to get the reinforcer).

During the sessions, all the Z-scores of the seven bands selected for each of the four channels were computed at each moment. The percentage of those scores within the specified range (±1.5 Z-score) was likewise computed in real time. The participants received reinforcement every time the percentage of Z-scores within the range was equal to or greater than the percentage requested by the software as a criterion for reinforcement. This was automatically calculated to guarantee a 50% reinforcement (Figure 4, Figure A1 and Figure A2 in Appendix B).

In order to produce feedback, a dimmer was overlapped on the screen where the films were projected. The dimmer became clear when the subject met the LZT-NF protocol criteria for receiving feedback and became opaque, preventing the patients from viewing the film, when they moved away from the criteria set out in the protocol (Figure 4).

All the participants underwent the same QEEG and were assessed with the CEC both at baseline and after the 10 sessions of NF training.

### 2.5. Statistical Analyses

We used the Mann–Whitney U test and Student’s *t*-test for continuous variables and chi-square (χ^2^) test for categorical variables. To further analyze the changes in QEEG metrics, the absolute Z-score for each participant’s wave was dichotomized (1 if |z-score| ≥ 1.5, and 0 otherwise). Thus, |z-score| ≥ 1.5 was categorized as “out of the normal range.” The primary outcome was a post-intervention change towards a decreased percentage of QEEG waves out of the normal range. The difference in the likelihood of change was analyzed with the odds ratio (OR) and a binary logistic model using a generalized estimating equation (GEE) with an independent correlation structure and robust standard errors. The GEE is a statistical approach that accounts for the correlation between measurements in clustered data (i.e., variables grouped by a cluster identification variable). Unlike ordinary logistic regression, which uses the maximum likelihood estimator, the GEE uses the quasi-likelihood function to estimate the parameters of the studied variables with repeated measures over time. The quasi-likelihood function specifies that the variance of the response variable depends on the mean without assuming a given distribution for the response variable [64]. One of its key features is that it allows the estimation of the correlation structure without having to assume a pre-specified structure [65]. We clustered the QEEG Z-scores by participants. Thus, clusters (i.e., individuals) are independent of one another, but the observations (i.e., waves) are assumed to be correlated within clusters. The GEE model tested the main effects of the group (1 = ni-APF; 0 = li-APF), waves (1 = out of the normal range; 0 = within the normal range), and group-by-wave interaction. The waves within the normal range in the li-APF group were used as the reference category. More details on the GEE model description for our approach are presented in Appendix C.

Pre–post differences in CEC-Total and CEC-Learning scores were analyzed with repeated measures ANOVA, with the group (li-APF/ni-APF) as a between-subjects factor and time (pre–post) as a within-subjects factor. The main effects and interactions were analyzed using Bonferroni’s correction. The Greenhouse–Geisser correction was used when a lack of sphericity was found in a repeated measures ANOVA. Statistical analyses were run with SPSS v26. The alpha level was set at 5%.

## 3. Results

**Figure 5:**
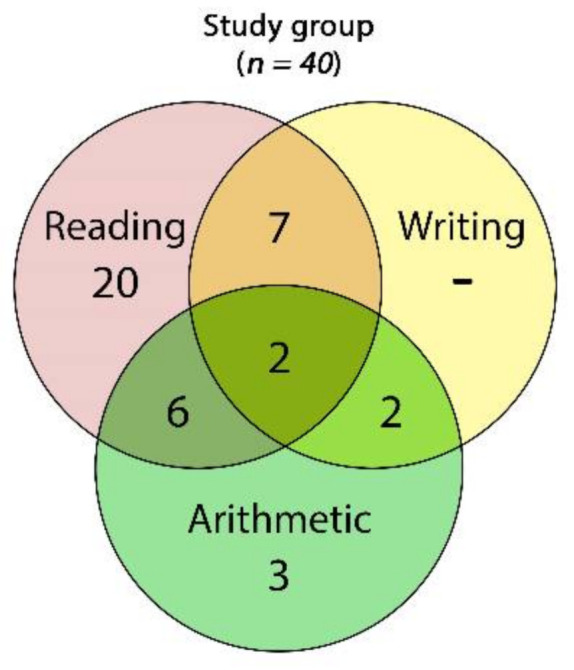
Venn diagram of the frequencies of cognitive impairments found across the whole study group: 2 children showed impairments in all of the three skills (reading, writing, and arithmetic), 6 had impairments in reading and arithmetic, 6 had reading and writing impairments, and 2 were impaired in writing and arithmetic.

From a pool of 45 potential participants, 5 girls were excluded because of missing data on the CEC. Eventually, data from 40 consecutive volunteer children and adolescents (35 boys) aged 12.07 years on average (SD = 1.63; range: 10–15) were analyzed. Most of the participants had, as single impairments, reading disabilities (20 participants, 50%), followed by a much smaller number of subjects with arithmetic impairments (3 participants, 7.5%), and we did not document any participants with writing disabilities only. Seventeen participants (42.5%) had two or more cognitive impairments (Figure 5).

**Figure 6:**
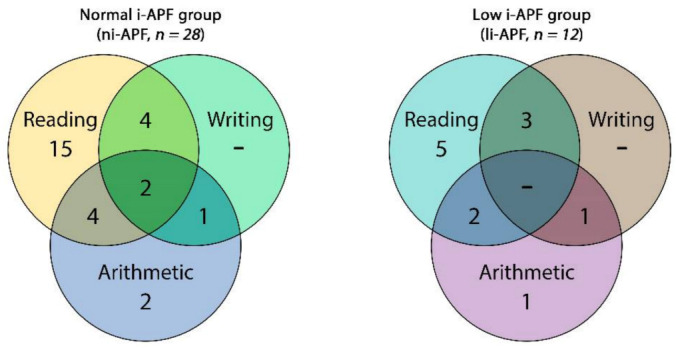
Venn diagram of the cognitive impairment frequencies of the in ni-APF vs. li-APF.

In the ni-APF group, we found 17 cases with only one disability—5 cases (53.6%) with reading disabilities and 2 cases (7.14%) with arithmetic disability—and none of the children from this group presented writing disabilities. The rest of the ni-APF subjects were shown to have combined disabilities: reading and arithmetic in 4 cases (14.3%); reading and writing in 4 cases (14.3%); writing and arithmetic in 1 case (3.6%); and reading, writing, and arithmetic in 2 cases (7.14%) (Figure 6).

In the li-APF group, there were 6 cases with only one disability: 5 (41.66%) with reading disabilities and 1 (8.33%) with an arithmetic disability; similarly, none of the children from this group presented writing disabilities only. As in the other group, the rest of the li-APF subjects had combined disabilities: reading and arithmetic in 2 cases (16.66%), reading and writing in 3 cases (25%), and writing and arithmetic in 1 case (8.3%).

No statistically significant differences between the ni-APF and li-APF groups were found in age (12.04 ± 1.45 vs. 12.33 ± 2.006 years, *p* = 0.760) or gender (25/3 vs. 10/2, *p* = 0.203). The i-APF mean in the li-APF group (8.54 ± 0.33 Hz) was significantly lower than that in the ni-APF group (10 ± 0.31 Hz, *p* = 0.000) (Appendix D, Table A2).

Pre-treatment, the absolute power Z-scores for each of the F3, F4, P3, and P4 locations showed no statistically significant differences, except for a higher mean theta band absolute power Z-score in P3 in the li-APF vs. ni-APF group (Appendix D, Table A3). After the treatment, a higher mean Hi-Beta band absolute power Z-score in F3 was found in the li-APF vs. ni-APF group (Appendix D, Table A3). Importantly, only after the LZT-NF intervention was there a statistically significant difference in both the CEC-Learning score means for li-APF vs. ni-APF (15.08 ± 1.93 vs. 11.46 ± 2.66, *p* = 0.000) and the CEC-Total score means for li-APF vs. ni-APF (43.75 ± 6.85 vs. 33.5 ± 7.23, *p* = 0.000) (Appendix D, Table A2).

The GEE model showed that the probability of change varied between the waves (OR = 16.87, standard error (SE) = 0.38, *p* < 0.001, 95% CI = 8.07–35.26), with the waves out of the normal range being more likely to change than the waves within the normal range. The differences in the waves’ probability of change between groups were not statistically significant (OR = 1.31, SE = 0.44, *p* = 0.538, 95% CI = 0.55–3.11) (Table 1).

**Figure 7:**
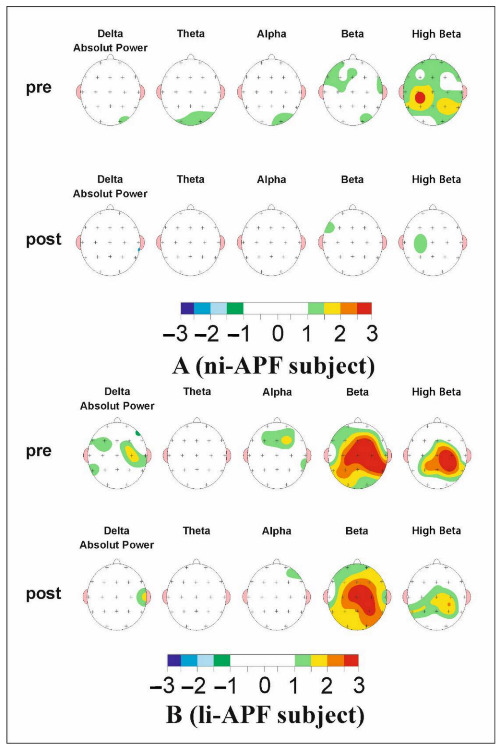
The map of a li-APF group subject’s (**A**) and a ni-APF group subject’s (**B**) pre (top)- and post (bottom)-intervention Z-scores. It can be seen how far each frequency band deviates from the norm (−1.5, +1.5 Z-scores) (the color scale for −3/+3 Z-scores under the maps indicates the deviations and whether they are positive or negative). An improvement in beta activity can be observed.

However, the group-by-wave interaction was statistically significant. Within the li-APF group, waves out of the normal range were more likely to change than waves within the normal range (OR = 2.39, SE = 0.39, *p* = 0.029, 95% CI = 1.09–2.25). Waves out of the range in the ni-APF group were more likely to change than waves within the normal range in the li-APF group (OR = 11.17, SE = 0.48, *p* < 0.001, 95% CI = 4.39–28.38). Waves within the normal range in the ni-APF group were less likely to change than waves within the normal range in the li-APF group (OR = 0.22, SE = 0.49, *p* = 0.003, 95% CI = 0.09–0.59) (Figure 7).

**Figure 8:**
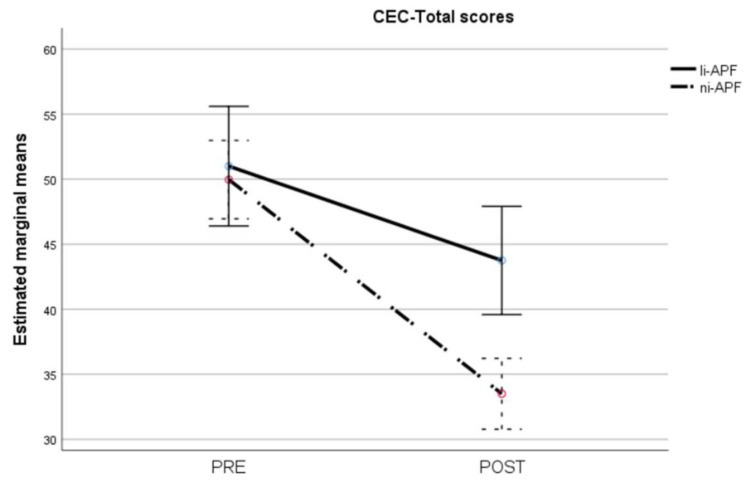
Pre-/post-intervention scores for cognitive and emotional tests (CEC-Total scores). The dotted line illustrates the results for the ni-APF group, and the continuous line represents the results for the li-APF group. Both groups were similar pre-intervention, but the ni-APF group achieved better results, further reducing the total scores in the CEC (with higher scores indicating problems of greater frequency and severity). Note that the initial scores for both groups overlap, but the final results do not.

The repeated measures ANOVA on the CEC-Total scores showed homoscedasticity (Box’s M = 2.42, *p* = 0.524). Multivariate analyses showed statistically significant effects of time (F = 151.97, *p* < 0.001), group (F = 5.51, *p* = 0.024), and group-by-time interaction (F = 22.94, *p* < 0.001). The group-by-time interaction is shown in Figure 1. There were no significant differences between the groups pre-test, whereas the ni-APF group showed a marked decrease in CEC-Total scores post-test (t = 4.17, *p* < 0.001) (Figure 8).

**Figure 9:**
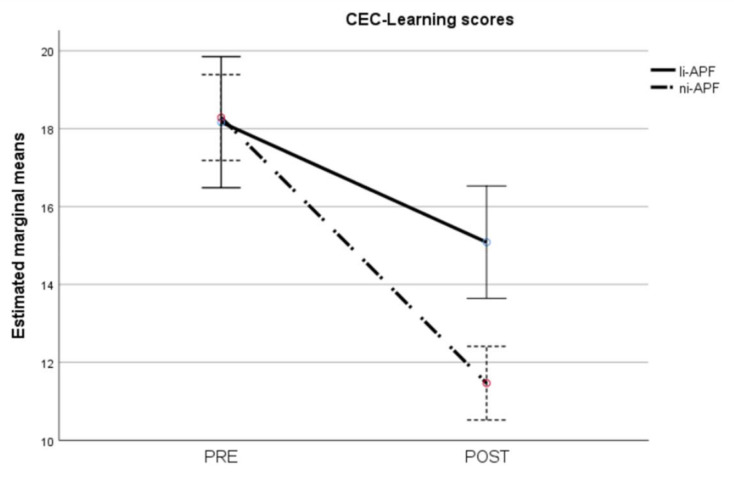
Pre-/post-intervention scores for cognitive and emotional test learning items. The results for the ni-APF group are illustrated with the dotted line, and the continuous line shows the results for the li-APF group. Both groups were similar pre-intervention, but the ni-APF group achieved better results, further reducing the learning scores in the CEC (with higher scores indicating problems of greater frequency and severity). As for the total scores, the initial scores for both groups overlap, but the post-treatment scores do not.

Regarding the CEC-Learning scores, the repeated measures ANOVA showed heteroscedasticity (Box’s M = 44.86, *p* = 0.012). Applying the Greenhouse–Geisser correction to the comparisons showed statistically significant effects of time (F = 160.57, *p* < 0.001), group (F = 4.35, *p* = 0.044), and group-by-time interaction (F = 22.87, *p* < 0.001). The group-by-time interaction is shown in Figure 9. As with the CEC-Total scores, there were statistically significant differences between the groups post-test (t = 4.25, *p* < 0.001) but not pre-test (t = −0.12, *p* = 0.905), with the ni-APF group showing the greatest improvement after the intervention.

## 4. Discussion

This work aimed to investigate whether the i-APF might be considered a potential moderator of the QEEG normalization after an LZT-NF intervention in children and adolescents with LDs. The gender prevalence data (boys vs. girls) are in line with other reports. More than two-thirds of school-aged children with LDs are males [1]. After the LZT-NF sessions, both the li-APF and ni-APF groups showed greater odds of moving impaired waves towards the norm. Our findings are consistent with the current literature related to learning disability conditions. Krigbaum and Wigton [38] used the progression of the mean Z-scores computed for each subject to study the normalization of the EEG in patients with ADHD. They separated the positive and negative Z-scores and found that after the LZT-NF intervention, there was a 90% normalization of the Z-scores. In another work on children and adults with ADHD, Groeneveld et al. [24] used Krigbaum and Wigton’s method but calculated the absolute values of the Z-scores and analyzed their tendency to be normalized after the LZT-NF intervention. They found a similar normalization rate in adults and children with ADHD. For LD children, recent studies with larger cohorts are scarce. Fernández et al. [21], for example, used a different approach—the theta/alpha ratio protocol—on a smaller sample and successfully optimized this procedure, comparing auditory with visual reinforcer efficiency to lower the theta/alpha ratio. Both of their subgroups exhibited relevant EEG maturation signs, highlighting the importance of neurofeedback training in these children.

Nonetheless, there are some important aspects to highlight in the specific phenotypes, such as li-APF patients. In our study, they did not improve their reading, arithmetic, and writing abilities as much as the ni-APF patients. The absolute power differences (higher for theta band in P3) were significantly higher in the posterior leads for the li-APF patients only in the QEEG evaluations pre-LZT-NF sessions. Previous studies on children with LDs with excess theta and low alpha suggest a maturation lag in their cognitive neural networks [3,10]. This might explain why after the LZT-NF intervention, the waves in the normal range for the li-APF group were more likely to move out of the norm than those for the ni-APF group. Therefore, based on the i-APF cutoff value and the CEC score results after LZT-NF sessions, we might consider li-APF patients as non-optimal responders addressing a notable gap in the literature. Then, an important observation that adds contribution to the current LD research is that the li-APF group significantly increased their Hi-Beta absolute power in F3 (Appendix D, Table A3). These changes could reflect a different or possibly prolonged, augmented, and/or compensatory response triggered by the mechanisms involved in synchronizing the cognitive networks to enhance cognitive performance. In a recent EEG-functional magnetic resonance imaging (EEG-fMRI) study on healthy younger adults, the positive feedback triggered a Hi-Beta power increase, which is believed to synchronize important areas and networks involved in learning from reward (ventral striatum, hippocampus, anterior temporal cortex, and posterior cingulate cortex) [66]. These observations could lead to further research in children with LD phenotypes to address the noted differences.

Our main finding highlights the i-APF as a useful biomarker for differentiating optimal and non-optimal responders to LZT-NF, in line with previous studies but on different medical conditions and different non-pharmacological treatments [18,67]. For instance, in tinnitus, Güntensperger et al. [67] used the i-APF to individualize an alpha frequency band of ±2 Hz around the peak frequency for each subject. Using an alpha/delta protocol NF, the authors were able to specifically train the configured frequency bands without changing the other bands. Their design resembles the LZT-NF technique’s goals but takes a different approach regarding the neurotherapy protocol. In this study, the responders reduced their tinnitus symptoms by increasing the individual alpha bands and decreasing delta (alpha/delta ratio improvement). The nonresponders did not report any changes, or in some cases, there was an increase in tinnitus symptoms. Likewise, Arns et al. [68], in a study on depressive patients treated with repetitive transcranial magnetic stimulation (rTMS), found the li-APF to be a marker of treatment for the nonresponders, and they found the same li-APF patients to also be nonresponders to drug treatment. Previous work from the same researchers also found that a personalized rTMS frequency (li-APF + 1 Hz) to modulate anterior li-APF (dorsolateral prefrontal cortex) function did not improve the clinical condition.

Each individual’s particular neurophysiology might explain the more satisfactory response in patients with ni-APF compared with those with li-APF. Individual EEG frequency band analysis revealed additional information about the neurophysiology of the brain’s electrical activity, showing different ranges for the same age according to individual variability [13,67,69]. This observation reinforces our idea that the particular neurophysiology of each individual affects the response to NF and that the i-APF could be a viable biomarker in this respect. Moreover, the LZT-NF technique could be a solution for optimally responding LD children based on i-APF categories.

With the LZT-NF, there is a tendency towards the normalization of the QEEG. In this regard, some authors [70,71] have pointed out that the deviation from the database can show differences with the norm, but the norm may not be optimal, so caution should be exercised [72]. No significant LZT-NF-related side effects have been reported in 15 years since the technique’s development [33,35,36]. Our previous reports [73,74], in several cases using the Wigton and Krigbaum method [38,39], and the current study further strengthen this idea.

This study has some limitations related to its nature (being a consecutive study, being gender unbalanced, and having no sham group). Although an unbalanced boys/girls ratio is common in previous works using NF [75,76,77], and sex seems not to be a modulator of NF learning performance [78], other authors have reported that girls with ADHD that remitted after NF treatment had shorter P300 latencies, an effect that was not observed in boys [79]. Thus, future research with larger and balanced samples should add the gender variable as a potential NF response moderator in adolescents with li-APF or ni-APF. Additionally, as the efficacies of both visual and audio reinforcers vs. placebo sham are acknowledged in the literature [1,9,80], including a control group with sham NF will support the idea that improvements in both brain waves and clinical symptoms are related to the NF training. The results reported in the present work need to be replicated in future gender-balanced, multicentric, randomized clinical control trials with larger cohorts, focusing on both optimal and especially non-optimal responder groups (li-APF), to gather more data about these phenotypes and to optimize the treatment outcomes.

Another observation is that there is a need for more exploratory approaches in neurofeedback protocols. First, the number of sessions of NF is slightly lower than the number of sessions reported in other works on children with LDs. For example, Fernández et al. reduced theta/alpha ratios using power training through 20 sessions [21], and Breteler et al. [81] trained power and coherence through 20 sessions. However, when using LZT-NF, it has been suggested that positive clinical outcomes can be achieved within an average of 10 to 20 sessions [33,37,82,83], as has been reported by our group in a patient with insomnia [74]. Then another limitation might be the wide age range in this study, but we based our approach on the i-APF maturation reports [20]. Although other neurofeedback studies used smaller samples, while our number of participants was higher than usual, this is an aspect that could be further improved to achieve greater statistical power and produce more generalizable results.

Future exploratory work using LZT-NF should include larger samples, study the differential effects with a variable number of sessions (e.g., 10, 20, or 30 sessions), and target narrower age ranges to control for possible influences from the subjects’ own maturation histories. In addition, an important direction for further exploratory studies is to understand the mechanisms involved in the li-APF subgroup of non-optimal responders for our LZT-NF approach using different advanced research designs; our study paves the way for this. For example, a study proposed by Martínez-Briones et al. [80] could be applied in our li-APF non-optimal responders for the LZT-NF. The authors used source localization methods, such as sLORETA (standardized low-resolution electromagnetic tomography analysis), to employ the power spectral density (PSD) analysis of the estimated primary currents at the source level in a specific cognitive task for LD children. Their methodology was based on a data-driven approach using the eigenvector centrality mapping (ECM) technique and an improved power method algorithm to select the specific regions of interest (ROI) involved in the specific task. Consequently, a global connectivity index for most of the subjects was computed. Using an unmixing signal algorithm, from the selected ROIs and further on the Fast Fourier Transform, the segments of unmixing signals were then transformed in the frequency domain. Eventually, a source frequency spectrum was selected for each ROI for each patient with each task condition. The final step involved an advanced statistical analysis (linear mixed model) of PSD performed to link each frequency with predictors, such as IQ, percentages of correct responses in cognitive tasks, and so forth. In this way, the authors identified over-recruitment in the slow bands of delta and theta associated with sustained concentration and attention, and under-recruitment in the left parietal gamma and left temporal beta bands associated with memory maintenance and response preparation. Further investigation of these findings with EEG–fMRI approaches could offer a solution for our li-APF subjects as non-optimal responders to LZT-NF and pave the way for innovative and more appropriate personalized neurofeedback protocols.

Our results might help clinicians interpret the results of NF interventions, as the i-APF could be useful for identifying optimal responders to LZT-NF in adolescents with LDs.

## 5. Conclusions

The i-APF metric reflects the patient’s neurophysiological individuality. It is a biomarker that should be considered as a moderator of the subject’s response to LZT-NF. Rather than finding responders and nonresponders, we found optimal responders for subjects with ni-APF and non-optimal responders for subjects with li-APF. This reinforces the idea that NF training can be optimized if the individual parameters of EEG activity are taken into consideration. Future research performed on larger cohorts should consider more in-depth analyses about each subject’s frequency bands’ individualization. Our results call for a more individualized approach to the NF treatment of LDs in children and adolescents.

## Figures and Tables

**Table 1:** Numbers of waves out of the normal range for the absolute power Z-scores (in absolute values) by group.

	Low i-APF Group (li-APF, *n* = 12)		Normal i-APF Group (ni-APF, *n* = 28)	
Waves	Pre	Post	Pre	Post
Abs Z < 1.5	257 (76.49%)	246 (73.21%)	519 (66.19%)	662 (84.44%)
Abs Z ≥ 1.5	79 (23.51%)	90 (26.79%)	265 (33.81%)	122 (15.56%)
Total	336	336	784	784

Numbers of absolute power Z-scores out of the normal range (in absolute values) by group were computed and are reported considering all the frequency bands. Absolute Z-score (Abs Z).

## Data Availability

Anonymized data are available upon request.

## Appendix A

**Table A1:** CEC-Learning items [54].

Item	Content	Score
11	Poor Short-Term Memory	0 1 2 3
14	List Learning Problems	0 1 2 3
29	Can’t Recall More Than One Request	0 1 2 3
30	Poor Maths Skills	0 1 2 3
31	Poor Reading Comprehension	0 1 2 3
42	Dyslexia	0 1 2 3
43	Reads Poorly	0 1 2 3
44	Poor Handwriting	0 1 2 3
48	Difficulty with Task Sequence	0 1 2 3
49	Difficulty Learning New Words	0 1 2 3

CEC-Learning items. 0 = no symptoms, 1 = mild, 2 = moderate, 3 = severe. Out of 49 items, we highlight the 10 CEC items related to learning, mathematics, reading, and writing (CEC-Learning). Learning disabilities (LDs) are diagnoses based on significantly lower performance in one or more tests measuring reading, arithmetic, or writing [2,3]. We analyzed 49 potential participants. Three patients were excluded because of the presence of mental disorders other than LDs (2 patients had ADHD and 1-ASD, and 1 patient had an IQ lower than 85). We enrolled 45 patients with LDs, but we excluded 5 girls with LDs because of missing data on the CEC scores. Reproduced with permission from Richard Soutar.

## Appendix B

The percentage of Z-scores within the specified range (±1.5 Z-score) computed in real-time. The reinforcement was received every time the percentage of the Z-scores was out of the range. It was automatically calculated to guarantee a 50% reinforcement.

## Appendix C

The GEE model is employed when there are multiple observations for each subject/cluster, estimating the variation within subjects/clusters. Consequently, GEE produces population-averaged estimates of the studied variables with their standard errors, 95% confidence intervals, and *p*-values. More specifically, the GEE method provides regression estimates for repeated data of explanatory variables, the so-called covariates considering the within-subject covariance matrix. It provides iteratively the best quasi-likelihood regression estimates $\left(\hat{\beta }\right)$ to describe the relationship between covariates ($X_{i}$) and non-normal responses ($Y_{i}$) (1). In our approach, we used the GEE model to predict the response (waves out of the normal range—1, waves within the normal range—0) correlating the observations within clusters (QEEG Z-scores by participants). We selected logistic regression to test the group’s main effects (1 = ni-APF, 0 = li-APF), waves (1 = out of normal range, 0 = within normal range), and group-by-wave interaction. In this case, we had a Bernoulli distribution because $\left(Y_{i\;}\right)$ is binomial, so the function g linking the responses ($Y_{i}$) to the parameters/covariates ($X_{i}$) is computed using the following formula [84,85]:

(A1) $$g\left(E\left(Y_{i}|X_{i}\right)\right)=\mathrm{logit}\left(p\right)=\log\left(\frac{p}{1-p}\right)={\hat{\beta }}_{0}+{\hat{\beta }}_{1}x_{1}+\ldots {\hat{\beta }}_{k}x_{k}$$

where *p* is the probability of decreasing the percentage of QEEG waves out of the normal range, $E\left(Y_{i}|X_{i}\right)=\mu_{i\;}$, $E\left(Y_{i}|X_{i}\right)\;\mathrm{is}$ the marginal expectation, and $\mu_{i}$ is the marginal mean; hence, considering $g\left(\mu_{i}\right)=X_{i}^{T}\hat{\beta }$, where ${\hat{\beta }}^{T}={\left({\hat{\beta }}_{1},\ldots ,{\hat{\beta }}_{k}\right)}^{T}$ is the k-dimensional vector of unknown regression coefficients [85].

(A2) $$E\left(Y_{ij}|x_{ij}\right)=\mu_{ij}=\frac{\exp\left(X_{i}^{T}\beta \right)}{1+\exp\left(X_{i}^{T}\beta \right)}$$

GEE solves the estimating Equation (A3):

(A3) $$\sum_{i=1}^{N}D_{i}^{T}V_{i}^{-1}\left(Y_{i}-\mu_{i}\right)=0$$

where $D_{i}=\partial \mu_{i}/\partial \beta$, $V_{i}=\mathrm{variance}\left(Y_{i}|X_{i}\right)=A_{i}^{1/2}M_{i}\left(\alpha \right)A_{i}^{1/2}$, $V_{i}$ is a working covariance matrix, Ai is a diagonal matrix with known variance function ν(µij), Mi(α) is a corresponding working correlation matrix presenting the within-subject dependence, and α is a generally unknown parameter. The working independence correlation matrix assumes no correlation among responses within subjects.

We employed the working independent correlation matrix, estimating the final variance for $\hat{\beta }$ as a linear combination of variance estimates produced by GEE:

(A4) $$\mathrm{var}\left(\hat{\beta }\right)=A^{-1}BA^{-1}$$

where $A=\sum_{i=1}^{N}D_{i}^{T}V_{i}^{-1}D_{i}$ and $B=\mathrm{variance}\left(\sum_{i=1}^{N}\;U_{i}\right)$ and $U_{i}=D_{i}^{T}V_{i}^{-1}\left(Y_{i}-\mu_{i}\right)$.

The sandwich-based estimator in the algorithm computes ($\mathrm{var}\left(\hat{\beta }\right)$) irrespective of the selected type for the working covariance matrix. It empirically calculates through the iterative process by substituting the estimate of $\hat{\beta }$ into Equation (A5) at each iteration and updates it for final estimate fixing the standard errors that might emerge from a mis-specified working covariance matrix:

(A5) $$V_{s}={\left(\sum_{i=1}^{N}D_{i}^{\prime }V_{i}^{-1}D_{i}\right)}^{-1}\left(\sum_{i=1}^{N}D_{i}^{\prime }V_{i}^{-1}\mathrm{Cov}(Y_{i})V_{i}^{-1}D_{i}\right){\left(\sum_{i=1}^{N}D_{i}^{\prime }V_{i}^{-1}D_{i}\right)}^{-1}$$

where $\mathrm{Cov}\left(Y_{i}\right)=E\left(Y_{i}-\mu_{i}\right){\left(Y_{i}-\mu_{i}\right)}^{T}$ [85].

## Appendix D

**Table A2:** I-APF, CEC-Total, and CEC-Learning scores (means and SD).

Parameter	li-APF Group (*n* = 28)		ni-APF Group (*n* = 12)		*p*-Value
**I-APF**	**Mean**	**SD**	**Mean**	**SD**	***0.000***
	8.54 Hz	0.33	10 Hz	0.31	
**CEC-Total**	**Mean**	**SD**	**Mean**	**SD**	***p*-Value**
Pre	51	6.88	49.96	8.24	0.850
Post	43.75	6.85	33.50	7.23	***0.000***
**CEC Learning**	**Mean**	**SD**	**Mean**	**SD**	***p*-Value**
Pre	18.17	1.95	18.29	3.18	0.965
Post	15.08	1.93	11.46	2.66	***0.000***

The *p*-value was computed in pairs, li-APF vs. ni-APF, for pre-treatment and then for post-treatment. The Mann–Whitney test was employed for all three parameters. Italic and bold indicate the value is significant.

**Table A3:** Z-scores of absolute power means (Ni-APF vs. Li-APF) pre- and post-treatment.

	Z-Scores	Ni-APF	Li-APF	*p*-Value
		Pre/Post	Pre/Post	Pre/Post
**F3**	Delta	0.70 (0.49)/0.62 (0.58)	0.72 (0.53)/0.62 (0.59)	0.545/0.825
	Theta	0.66 (0.61)/0.58 (0.38)	0.80 (0.39)/0.92 (0.70)	0.140/0.121
	Alpha	0.92 (0.63)/0.73 (0.50)	0.80 (0.49)/0.86 (0.67)	0.734/0.723
	Beta-1	1.16 (0.95)/0.67 (0.62)	0.71 (0.68)/0.98 (0.82)	0.101/0.626
	Beta-2	1.16 (0.73)/1.02 (0.55)	0.77 (0.56)/0.98 (0.77)	0.152/0.757
	Beta-3	1.23 (0.81)/0.92 (0.55)	1.10 (0.71)/1.34 (0.81)	0.669/0.087
	Hi-Beta	1.52 (0.82)/1.11 (0.73)	1.90 (1.23)/2.05 (1.19)	0.479/***0.007***
**F4**	Delta	0.86 (0.62)/0.54 (0.35)	0.72 (0.47)/0.61 (0.60)	0.690/0.768
	Theta	0.70 (0.68)/0.51 (0.33)	0.76 (0.63)/0.68 (0.64)	0.605/0.848
	Alpha	0.89 (0.68)/0.79 (0.52)	0.87 (0.73)/0.75 (0.47)	0.926/0.813
	Beta-1	1.29 (0.97)/1.04 (0.79)	0.85 (0.73)/0.84 (0.81)	0.125/0.215
	Beta-2	1.16 (0.89)/0.95 (0.69)	1.00 (0.62)/0.84 (0.77)	0.757/0.425
	Beta-3	1.21 (0.79)/0.95 (0.53)	1.16 (0.64)/1.20 (0.75)	0.976/0.443
	Hi-Beta	1.49 (0.88)/1.00 (0.86)	1.49 (1.02)/1.60 (1.42)	0.906/0.148
**P3**	Delta	0.82 (0.82)/0.70 (0.54)	0.89 (0.68)/0.71 (0.53)	0.425/0.976
	Theta	0.77 (0.68)/0.54 (0.32)	0.76 (0.35)/0.67 (0.59)	0.215/0.768
	Alpha	1.02 (0.63)/0.85 (0.54)	0.96 (0.70)/0.86 (0.69)	0.637/0.701
	Beta-1	1.33 (0.91)/1.03 (0.61)	0.79 (0.86)/0.86 (0.72)	0.070/0.262
	Beta-2	1.50 (0.74)/1.07 (0.55)	0.96 (0.81)/1.83 (2.36)	0.063/0.434
	Beta-3	1.62 (0.78)/1.19 (0.59)	1.14 (0.80)/1.23 (0.66)	0.090/1.00
	Hi-Beta	1.94 (1.10)/1.29 (0.61)	1.99 (0,99)/1.47 (0.89)	0.779/0.352
**P4**	Delta	0.64 (0.43)/0.61 (0.50)	0.65 (0.49)/0.79 (0.50)	0.941/0.294
	Theta	0.62 (0.60)/0.59 (0.37)	0.87 (0.33)/0.74 (0.58)	***0.016***/0.516
	Alpha	0.89 (0.57)/0.80 (0.48)	1.00 (0.53)/1.64 (2.11)	0.479/0.148
	Beta-1	1.27 (0.91)/1.11 (0.76)	0.82 (0.84)/0.80 (0.97)	0.128/0.152
	Beta-2	1.47 (0.88)/1.20 (0.73)	0.98 (0.75)/1.11 (0.80)	0.092/0.658
	Beta-3	1.58 (0.83)/1.20 (0.64)	1.14 (0.74)/1.30 (0.63)	0.125/0.690
	Hi-Beta	1.82 (1.00)/1.39 (0.80)	1.79 (0.85)/1.72 (0.89)	0.918/0.256

Absolute power Z-score means and SD. The *p*-value was computed in pairs, ni-APF/li-APF, for pre-treatment and then for post-treatment. The Mann–Whitney test was employed for all parameters. Statistical significance was considered for *p* < 0.05. Note the higher Z-score mean for the P4 theta band absolute power for li-APF vs. ni-APF. Post-treatment, the analysis highlighted differences only in Hi-Beta, with a higher Z-score mean in F3 lead in li-APF vs. ni-APF. A significant difference to note is the increase in the Hi-Beta Z-score mean after treatment in li-APF children in F3 lead compared with ni-APF children, revealing an opposite behavior (a decrease in Hi-Beta Z-score mean post-treatment). Italic and bold indicate the value is significant.
